# Perioperative liberal versus restrictive fluid strategies and postoperative outcomes: a systematic review and metanalysis on randomised-controlled trials in major abdominal elective surgery

**DOI:** 10.1186/s13054-021-03629-y

**Published:** 2021-06-11

**Authors:** Antonio Messina, Chiara Robba, Lorenzo Calabrò, Daniel Zambelli, Francesca Iannuzzi, Edoardo Molinari, Silvia Scarano, Denise Battaglini, Marta Baggiani, Giacomo De Mattei, Laura Saderi, Giovanni Sotgiu, Paolo Pelosi, Maurizio Cecconi

**Affiliations:** 1 Department of Anaesthesia and Intensive Care Medicine, Humanitas Clinical and Research Center – IRCCS, Via Alessandro Manzoni, 56, 20089 Rozzano, MI Italy; 2 Department of Biomedical Sciences, Humanitas University, Pieve Emanuele, MI Italy; 3 Anaesthesia and Intensive Care, IRCCS for Oncology and Neuroscience, San Martino Policlinico Hospital, Genoa, Italy; 4 Department of Surgical Sciences and Integrated Diagnostic (DISC), University of Genoa, Genoa, Italy; 5 Anesthesia and Intensive Care Medicine, Maggiore Della Carità University Hospital, Novara, Italy; 6 Anesthesia and Intensive Care Medicine, Azienda Sanitaria Universitaria Integrata Udine, Udine, Italy; 7 Clinical Epidemiology and Medical Statistics Unit, Department of Medical, Surgical and Experimental, University of Sassari, Sassari, Italy

**Keywords:** Liberal, Restrictive, Fluid therapy, Postoperative complications, Postoperative mortality

## Abstract

**Background:**

Postoperative complications impact on early and long-term patients’ outcome. Appropriate perioperative fluid management is pivotal in this context; however, the most effective perioperative fluid management is still unclear. The enhanced recovery after surgery pathways recommend a perioperative zero-balance, whereas recent findings suggest a more liberal approach could be beneficial. We conducted this trial to address the impact of restrictive *vs.* liberal fluid approaches on overall postoperative complications and mortality.

**Methods:**

Systematic review and meta-analysis, including randomised controlled trials (RCTs). We performed a systematic literature search using MEDLINE (via Ovid), EMBASE (via Ovid) and the Cochrane Controlled Clinical trials register databases, published from 1 January 2000 to 31 December 2019. We included RCTs enrolling adult patients undergoing elective abdominal surgery and comparing the use of restrictive/liberal approaches enrolling at least 15 patients in each subgroup. Studies involving cardiac, non-elective surgery, paediatric or obstetric surgeries were excluded.

**Results:**

After full-text examination, the metanalysis finally included 18 studies and 5567 patients randomised to restrictive (2786 patients; 50.0%) or liberal approaches (2780 patients; 50.0%). We found no difference in the occurrence of severe postoperative complications between restrictive and liberal subgroups [risk difference (95% CI) = 0.009 (− 0.02; 0.04); *p* value = 0.62; *I*_2_ (95% CI) = 38.6% (0–66.9%)]. This result was confirmed also in the subgroup of five studies having a low overall risk of bias. The liberal approach was associated with lower overall renal major events, as compared to the restrictive [risk difference (95% CI) = 0.06 (0.02–0.09); *p* value  = 0.001]. We found no difference in either early (*p* value  = 0.33) or late (*p* value  = 0.22) postoperative mortality between restrictive and liberal subgroups

**Conclusions:**

In major abdominal elective surgery perioperative, the choice between liberal or restrictive approach did not affect overall major postoperative complications or mortality. In a subgroup analysis, a liberal as compared to a restrictive perioperative fluid policy was associated with lower overall complication renal major events, as compared to the restrictive.

**Trial Registration:**

CRD42020218059; Registration: February 2020, https://www.crd.york.ac.uk/prospero/display_record.php?RecordID=218059.

**Supplementary Information:**

The online version contains supplementary material available at 10.1186/s13054-021-03629-y.

## Introduction

A worldwide effort aims to reduce postoperative complications[1], which are recognised as partially preventable events affecting long-term morbidity and impacting health and financial systems [2, 3]. Several perioperative strategies have been proposed to optimise intraoperative management and postoperative care [4]. Among them, perioperative fluid therapy is a core concept. The ideal perioperative approach has been debated for decades, having the crucial role of balancing oxygen supply and demand, maintaining fluid and electrolyte homeostasis and avoiding inadequate tissue perfusion and fluid overload [5–12].

The most effective perioperative fluid management is still unclear [13–15]. It has been classified as restrictive (< 1.75 L per day), balanced (1.75 to 2.75 L per day) and liberal (> 2.75 L per day)[16]. However, the literature provides different, somewhat overlapping definitions (*i.e.* from 1.0 to 2.7 L for restrictive, compared with 2.8 to 5.4 L for liberal fluid regimens) [17] and conflicting evidence [13–15]. The enhanced recovery after surgery (ERAS) pathways to support early recovery among patients undergoing major surgery recommend a restrictive approach aiming at the perioperative “zero-balance”[13]. In contrast, recent findings suggest that excessively restrictive approaches could be detrimental, indicating that a moderately liberal fluid regimen (i.e. positive fluid balance of 1 to 2 L at the end of surgery) might be the best approach[14].

Interestingly, a recent large randomised controlled trial (RCT) assigning 2983 patients to either zero-balance or liberal strategy showed comparable disability-free survival outcome, although the zero-balance approach was associated with a higher rate of acute kidney injury [16]. As a matter of fact, this single study enrolled more patients than several previous RCTs regarding this topic, insofar, partially challenging previous results [18–20].

Therefore, we conducted an up-to-date meta-analysis of RCTs to assess the association between restrictive and liberal strategies and major adverse surgical outcomes in elective surgery.

Secondarily, we appraised the association between restrictive and liberal approaches on perioperative mortality, predefined postoperative organ-related complications and hospital length of stay. Finally, we stratified the studies according to preoperative severity score and the reported rate of complications [*i.e.* American Society of Anaesthesiologists (ASA) physical status score].

## Materials and methods

We adhered to the *Preferred Reporting Items for Systematic Reviews and Meta-Analysis—Protocols* (PRISMA-P) guidelines [21] (Additional file 1: Table 1) and the study protocol was registered [(*International Prospective Register of Systematic Reviews*—PROSPERO (CRD42020218059)].

### Data sources and search strategy

A systematic literature search was performed by using the following databases to identify relevant studies in indexed scientific journals: MEDLINE (via Ovid), EMBASE (via Ovid) and the Cochrane Controlled Clinical trials register, by using the terms: [(“liberal” OR “restrictive” OR “zero-balance” AND (“surgery”/exp OR surgery)] with filters for humans, age (< 18 years), language (English) and time of publication (1 January 2000 to 31 December 2019).

We included RCTs 1) enrolling adult patients undergoing elective abdominal surgery; 2) comparing two different regimes of fluid administration defined as “restrictive” and “liberal” or “conventional” or “standard”; 3) starting the study protocol intraoperatively; 4) reporting postoperative complications or mortality or as primary or secondary outcomes.

The restrictive approach was defined as a modality of perioperative (*i.e.* intraoperative and during the first 24 h after surgery) treatment employing a specific and predefined treatment protocol to obtain in one of the enrolled populations an overall negative or zero fluid balance, as compared to the other. Accordingly, trials showing no statistically significant difference in the overall fluid intake or balance, between restrictive and liberal subgroups, were also excluded.

Studies involving cardiac, non-elective surgery, paediatric or obstetric surgeries were excluded. We also excluded editorials, commentaries, letters to the editor, opinion articles, reviews, meeting abstracts and original articles lacking abstract and/or quantitative details, or those enrolling less than 15 patients in each subgroup.

The references of all included papers, review articles, commentaries and editorials on this topic were also reviewed to identify other studies of interest missed during the primary search.

### Data extraction and quality assessment

Three couples of examiners (S.S., E.M., D.B., F.I., M.B., G.D.M.) independently evaluated titles and abstracts. The articles were then subdivided into three subgroups: “included” and “excluded” (if the two examiners agreed with the selection) or “uncertain” (in case of disagreement). In the case of “uncertain” classification, discrepancies were resolved by further examination performed by two expert authors (A.M. and C.R.). We used a standardised electronic spreadsheet (Microsoft Excel, V 14.4.1; Microsoft, Redmond, WA) to extract the data from all included studies, recording trial characteristics (the complete data reporting sheet is provided in Additional file 1: Table 2). When necessary, the included studies’ corresponding authors were contacted to obtain missing data related to trial demographics, methods and outcomes.

### Risk of bias assessment in the included studies

Two examiners (L.C. and D.Z.) independently assessed the internal validity of the included studies and discrepancies were resolved by a third senior author (A.M. or C.R.), by using the RoB 2 (a revised Cochrane Collaboration’s risk of bias tool for randomised trials) [22]. The RoB 2 considers five bias domains: 1) the randomisation process; 2) the deviations from intended interventions; 3) missing outcome data; 4) measurement of the outcome; 5) selection of the reported results. Finally, the overall risk of bias was calculated and, accordingly, studies were included in either high-risk/ some concerns /low-risk groups.

### The strength of the body of evidence

The strength of the body of evidence was assessed according to the *Grading of Recommendations, Assessment, Development and Evaluations* (GRADE) evidence system[23].

### Outcomes definitions

Our primary outcome was to appraise the effect of restrictive *vs* liberal approaches on the overall rate of major complications. This outcome was assessed both considering all the studies reporting it, and those in the subgroup having a low-risk of bias, according to the RoB 2 scale.

Secondary outcomes were: to evaluate the association between restrictive and liberal approaches on perioperative early (≤ 30 postoperative days) and late (i.e. > 30 postoperative days) mortality and predefined postoperative major complications: renal (*i.e.* worsening of renal function, according to the trial definition); cardiovascular [*i.e.* pulmonary not infective complications, cardiac ischemic dysfunction/failure, cardiac arrhythmias; acute neurological events); infections (*i.e.* all infective complications reported, including the occurrence of either sepsis or septic shock). We also evaluated the length of hospital stay and stratified the studies according to the enrolled patients’ ASA classification.

### Statistical analysis

Statistical analysis was conducted on the summary statistics described in the selected articles (e.g. means, medians, proportions) and, therefore, the statistical unit of observation for all the selected variables was the single study and not the patient. Descriptive statistics of individual studies used different statistical indicators for central tendency and variability, such as means and standard deviations, whereas absolute and relative frequencies were adopted for qualitative variables. To show one single indicator for the quantitative variables we collected means with standard deviations (SD) or medians and inter-quartile ranges (IQR) were used, as appropriate.

The metanalysis included only those studies reporting mortality and rate of complications, according to the definition adopted in each study and expressed as rate with respect of the enrolled populations. For ASA subgroup analysis, we grouped studies with at least 50% of the included population classified as ASA I/II, compared to those having at least 50% of the included population classified as ASA III/IV. We, finally, stratified the studies according to three tertiles of the overall amount of perioperative fluid given (day 0 and day 1) and the rate of major events.

We assessed publication bias using a funnel plot for the considered outcomes. Statistical heterogeneity and inconsistency were measured using Q and *I*^2^ tests and were deemed to be significant when *p* < 0.1 and *I*^2^ > 50%. ﻿According to heterogeneity, random or fixed-effect models were used to perform metanalysis. According to Higgins et al. [24], *I*^2^ values around 25, 50 and 75% were considered representing, respectively, low, moderate and severe statistical inconsistency.

The statistical software STATA®13 (StataCorp, College Station, TX, USA) was used to perform all the computations.

## Results

**Fig. 1:**
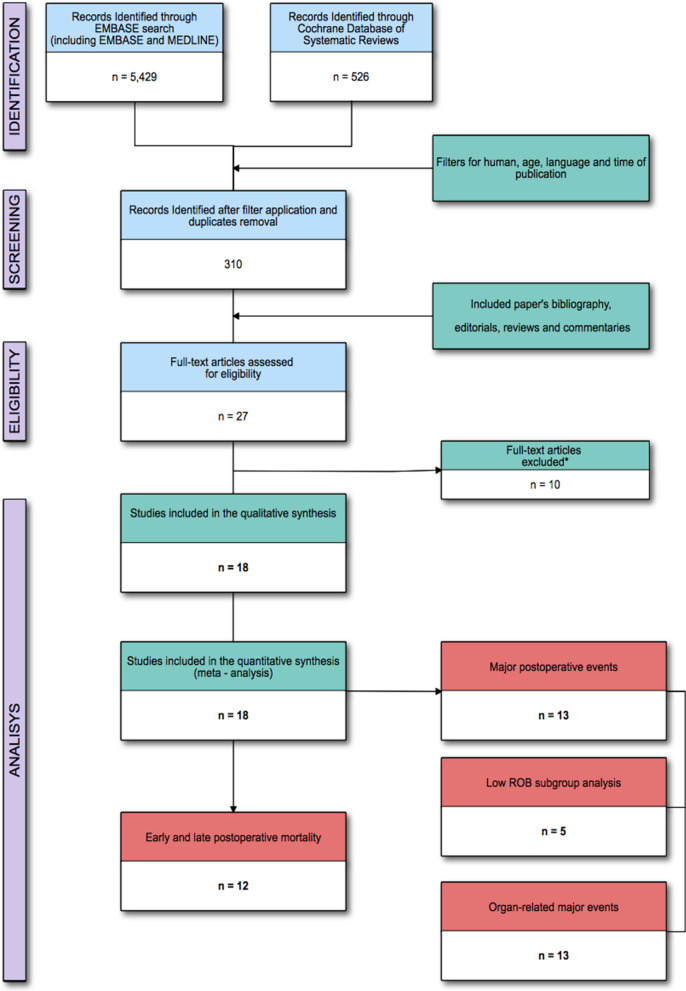
Flow of the studies. * = Not fitting eligibility criteria full-text articles excluded are reported in Additional file 1: Table 3. ROB, risk of bias

We identified 26 potentially eligible studies after the title and abstract assessment (Fig. 1 and Additional file 1: Table 3). After full-text examination, the metanalysis finally included 18 studies and 5567 patients (male/female ratio 1.1:1) randomised to restrictive (2786 patients; 50.0%) and liberal approaches (2780 patients; 50.0%). All the studies, except one [25], have been conducted on non-obese patients, the vast majority being scheduled for laparotomic (3925 patients, 70.5%) surgery. All the studies, except two [25, 26], were conducted on patients with a median age > 60 years old (Table 1). Interestingly, in 5 studies (27.7%), including the largest one [16], the perioperative fluid administration algorithm was guided by the optimisation of predefined or flow haemodynamic pressure variables, by means of a goal-directed therapy [16, 25, 27–29] (Additional file 1: Table 4).

**Table 1:** Summary of the included studies: general characteristics of the enrolled population

References	Year	Pt. analysed *n* (%)	RES *n* (%)	LIB *n* (%)	Age (years)		BMI (kg/m^2^)		ASA I, *n* (%)		ASA II, *n* (%)		ASA III–IV, *n* (%)	
					RES	LIB	RES	LIB	RES	LIB	RES	LIB	RES	LIB
Brandstrup [46]	2003	141	69 (48.9)	72 (51.1)	64	69	25	25	34 (49.3)	32 (44.4)	33 (47.8)	39 (54.2)	2 (2.9)	1 (1.4)
Kabon [26]	2005	253	124 (49)	129 (51)	53	52	NA	NA	13 (10.5)	9 (7.0)	81 (65.3)	86 (66.7)	30 (24.2)	34 (26.4)
Nisanevich [28]	2005	152	77 (50.7)	75 (49.3)	63	59	26	25	15 (19.5)	19 (25.3)	42 (54.5)	37 (49.3)	20 (26)	19 (25.3)
Holte [39]	2007	32	16 (50)	16 (50)	74	77	26	24	5 (31.2)	3 (18.8)	2 (12.5)	5 (31.2)	8 (50)	9 (56.2)
Muller [37]	2009	151	76 (50.3)	75 (49.7)	62	59	24	26	2 (2.6)	3 (4.0)	50 (65.8)	54 (72)	24 (31.6)	18 (24)
Futier [36]	2010	70	36 (51.4)	34 (48.6)	62	60	25	24	19 (52.8)	20 (58.8)	9 (25)	6 (17.6)	8 (22.2)	7 (20.6)
Gao [30]	2012	179	93 (52)	86 (48)	72	73	22	22	33 (35.5)	32 (37.2)	27 (29)	24 (27.9)	33 (35.5)	30 (34.9)
Matot [25]	2012	107	52 (48.6)	55 (51.4)	40	42	44	43	0 (0.0)	0 (0.0)	33 (63.5)	38 (69.1)	19 (36.5)	17 (30.9)
Abraham [38]	2012	161	82 (50.9)	79 (49.1)	68	69	25	25	19 (23.1)	21 (26.6)	49 (59.7)	53 (67.1)	11 (14.4)	8 (10.1)
Kalyan [32]	2013	239	121 (50.6)	118 (49.4)	70	70	26	26	28 (23.1)	17 (14.4)	70 (57.9)	75 (63.6)	23 (19.0)	26 (22.0)
Lavu [33]	2014	250	131 (52.4)	128 (51.2)	67	68	27	25	NA	NA	NA	NA	83 (63.4)	84 (65.6)
Hong-Ying [49]	2014	185	89 (48.1)	96 (51.9)	65	65	22	22	29 (32.6)	30 (31.3)	37 (41.6)	41 (42.7)	23 (25.8)	25 (26)
Piljic [34]	2015	60	30 (50)	30 (50)	69	69	25	25	NA	NA	NA	NA	NA	NA
van Samkar [47]	2015	54	30 (55.6)	24 (44.4)	NA	NA	NA	NA	NA	NA	NA	NA	NA	NA
Grant [31]	2016	330	166 (50.3)	164 (49.7)	65	65	26	27	7 (4.2)	6 (3.7)	88 (53)	90 (54.9)	71 (42.8)	68 (41.5)
Kassim [29]	2016	50	25 (50)	25 (50)	67	66	NA	NA	NA	NA	NA	NA	NA	NA
Myles [16]	2018	2983	1490 (49.9)	1493 (50.1)	66	66	NA	NA	25 (1.7)	21 (1.4)	542 (36.4)	540 (36.2)	923 (61.9)	932 (62.4)
Wuethrich [35]	2014	166	83 (50.0)	83 (50.0)	69	68	24	24	0 (0.0)	0 (0.0)	52 (63)	46 (55)	31 (37)	37 (45)

*N*: number of patients; NA: data not available; ASA: American Society of Anaesthesiologists physical status; RES/LIB refers to those patients who received restrictive or liberal strategy, respectively. Age and body mass index (BMI) columns report the mean/median values of the LIB/RES populations recorded in each study

**Fig. 2:**
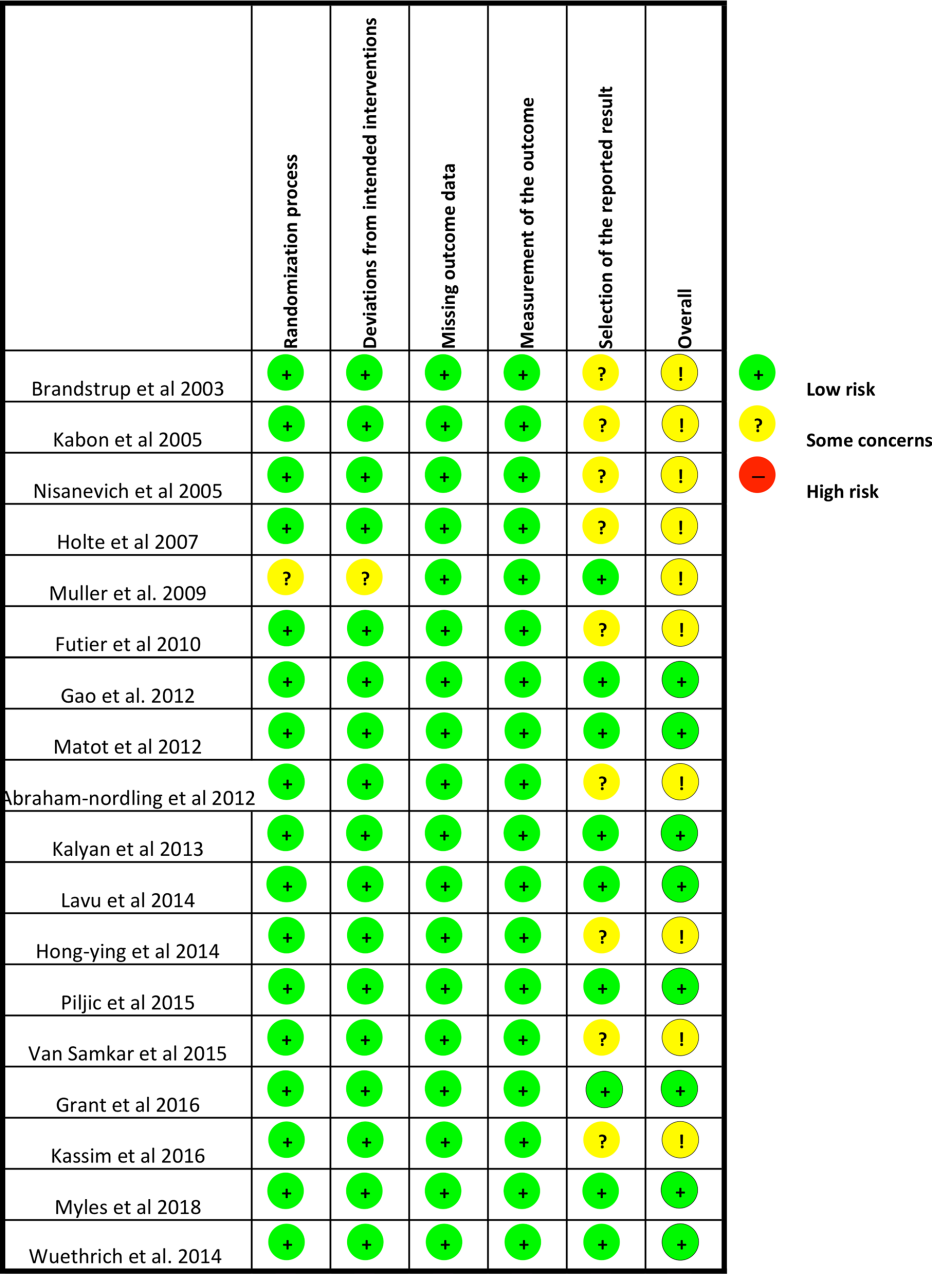
The internal validity of the included studies was assessed by two expert authors, by using the Rob2: a revised Cochrane Collaboration’s risk of bias tool for randomised trials [22]

The risk of bias assessment reported: “low risk” for 8 (44.4%) and “some concerns” for 11 10 (55.5%) of the included studies, mostly (9 of these 11; 81.8%) related to the selection of the reported results (Fig. 2).

Following the GRADE system, the quality of evidence was defined as high for 8 studies [16, 25, 30–35] and moderate for all the others.

### Perioperative fluid administration, balance and weight gain

Intraoperatively, patients in the restrictive subgroup overall received a median (IQR) of 1925 (1482–2470) of fluids, as compared to 3878 (3000–4400) of the liberal subgroup. On day 0 (*i.e.* considering the overall amount of fluids received on the day of the operation, including intraoperative fluid therapy), patients in the restrictive subgroup overall received a median (IQR) of 2341 ml (1635–3530) of fluids, as compared to 4,350 ml (3095–5326) in the liberal subgroup. Finally, considering day 0 and day 1, a median of 3617 (2897–5291) of fluids was administered to the restrictive subgroups, as compared to 5820 (5038–7000) to the liberal subgroups (Table 2 and Additional file 1: Table 4).

**Table 2:** Summary of the included studies: fluid infused on day 0 and day 1

References	IO COLLOIDS (%)		COLLOIDS IO (ml)		CRYSTALLOIDS IO (ml) CRYSTALLOIDS IO (ml)		TOTAL FLUIDS IO (ml) TOTAL FLUIDS IO (ml)		FLUIDS DAY 0 (IO + PO) (ml)		FLUIDS DAY 1 (ml)	
	LIB	RES	LIB	RES	LIB	RES	LIB	RES	LIB	RES	LIB	RES
Brandstrup [46]	NA	NA	NA	NA	NA	NA	NA	NA	5388 (2700–11,083)	2740 (1100–8050)	1500 (0–6000) D1	500 (0–5000) D1
Kabon [26]	0	0	0	0	3900 (1900)	2500 (1300)	NA	NA	5000 (400)	3100 (300)	NA	NA
Nisanevich [28]	NA	NA	NA	NA	NA	NA	3878 (1170)	1408 (946)	3878 (1170)	1408 (946)	2012 (475) D1	2170 (476) D1
Holte [39]	11.3	30.4	500 (350–750)	500 (341–850)	3900 (2722–6500)	1140 (580–1500)	NA	NA	5050 (3563–8050	1640 (935–2250)	NA	NA
Muller [37]	NA	NA	NA	NA	NA	NA	2950 (1600–6400)	1925 (1100–6700)	2950 (1600–6400)	1925 (1100–6700)	2700 (1800–9100) D1	1200 (3900–9900) D1
Futier [36]	5.6	25.2	316 (311)	854 (547)	5266 (1340)	3040 (769)	NA	NA	5588 (1463)	3380 (1114)	NA	NA
Gao [30]	40.6	13.5	1240 (410)	210 (300)	1450 (310)	1320 (220)	3050 (800)	1555 (410)	3050 (800)	1555 (410)	2730 (560) D1	2100 (340) D1
Matot [25]	NA	NA	NA	NA	NA	NA	3300 (1500–14,000)	1325( 480–3100)	NA	NA	NA	NA
Abraham [38]	0	0	0 (0–500)	0 (0–200)	2500 (2000–3070)	575 (452–800)	NA	NA	5775 (5050–6700)	3050 (2662–3328)	NA	NA
Kalyan [32]	NA	NA	NA	NA	NA	NA	2033 (1576–2500)	1000 (690–1500)	NA	NA	3315(2645–3894) D1	1944(1354–2515) D1
Lavu [33]	0	0	0	0	6694	4812	6694	4812	8789	5731	3312	3042
Hong-Ying [49]	NA	NA	NA	NA	NA	NA	3110	1620	NA	NA	2750 (570) D1	2090 (360) D1
Piljic [34]	25.9	15.9	675 (513)	315 (409)	1923 (593)	1662 (655)	2598 (505)	1977 (528)	5017	4038	2613 (549) D1	2006 (549) D1
van Samkar [47]	22.7	40.0	1000 (0.6)	1400 (0.6)	3400 (2500–6000)	2100 (1600–2500)	NA	NA	NA	NA	2500	2500
Grant [31]	9.2	15.9	363 (0–4000)	390 (0–2500)	3563 (1050–7550)	2050 (650–5130)	NA	NA	2151 (1030–4856)	1178 (550–4791)	2577 (1650–6500) D1	1436 (1000–4245) D1
Kassim [29]	20.4	50.0	920 (206)	1219 (268)	3586 (473)	1219 (140)	NA	NA	NA	NA	NA	NA
Myles [16]	14.2	22.9	500 (400–1000)	500 (250–800)	3000 (2100–3850)	1677 (1173–2294)	NA	NA	4300	2737	3100	2056
Wuethrich [35]	0	0	0	0	4300 (2800–6200)	1700 (700–4000)	4300 (2800–6200)	1700 (700–4000)	NA	NA	2050 (1000–4100)	2100 (800–4000)

Data are reported with the appropriate ranges, according to those present in the included studies. Data without ranges are obtained from data reported in the study. LIB, liberal; RES, restrictive; IO, intraoperative; PO, postoperative (including the whole period after the operation spent either in recovery room or intensive care unit); DAY 1, first day after the operation; NA, data not available. The first column reports the overall rate of intraoperative rate of colloids administration, as compared to the overall intraoperative amount of fluids

Only two studies reported the mean postoperative overall fluid balance among restrictive and liberal subgroups [16, 33]. Nine studies (50.0%) did not report postoperative weight gain [25, 26, 29–31, 33, 34, 36, 37], moreover, in Myles et al.’s study, data regarding this variable were missing for 1,036 (69.5%) patients in the restrictive fluid group and 999 (66.9%) in the liberal fluid group [16].

Five studies (27.7%) [16, 28, 35, 38, 39] reported a median (IQR) weight gain of 0.3 kg (− 0.1 to 0.65) and 2.0 kg (1.7–2.6) in restrictive and liberal groups on postoperative day 1, respectively.

### Primary outcome: rate of major complications

**Fig. 3:**
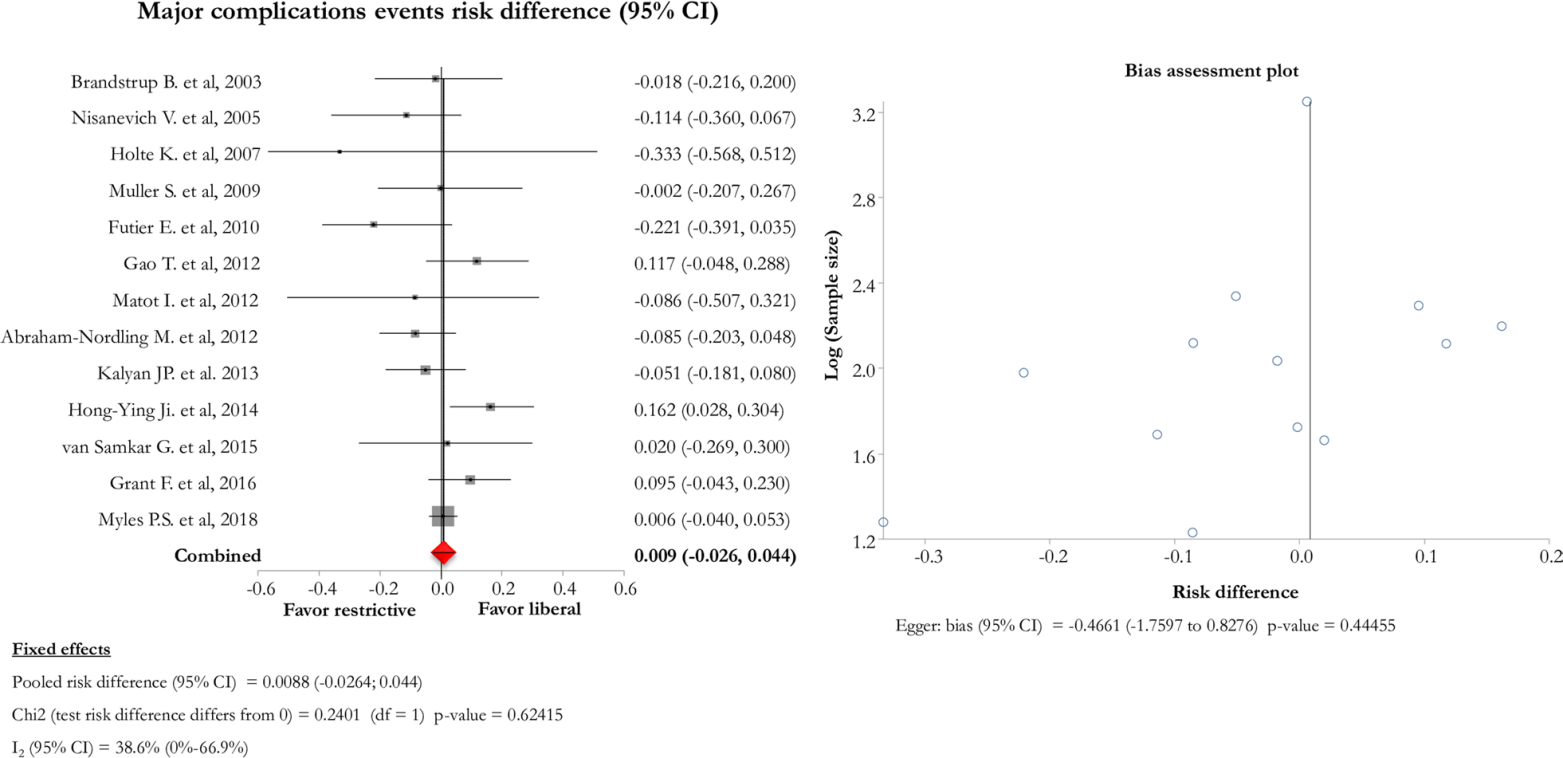
Forest and bias assessment plots of postoperative major events

Pooling data from the 13 studies, we found no difference in the occurrence of major postoperative complications between restrictive and liberal subgroups [pooled risk difference (95% CI) = 0.009 (− 0.02; 0.04); Chi_2_ = 0.24; *p* value  = 0.62; *I*_2_ (95% CI) = 38.6% (0–66.9%)] (Fig. 3). In the subgroup of five studies [16, 25, 30–32] reporting the outcome of major postoperative events and having a low overall risk of bias, we found no difference between restrictive and liberal subgroups [pooled risk difference (95% CI) = 0.013 (−0.02; 0.05); Chi_2_ = 0.42; *p* value  = 0.51; *I*_2_ (95% CI) = 1.0% (0–64.5%)].

### Secondary outcomes

#### Postoperative mortality

We found no difference in either early [data pooled from the 10 studies—pooled risk difference = − 0.005 (95% CI − 0.016 to 0.005); Chi_2_ = 0.95; *p* value  = 0.33; *I*_2_ = 0% (95% CI 0% to 52.7%)] or late [data pooled from the 8 studies—pooled risk difference = 0.005 (95% CI − 0.003 to 0.012); Chi_2_ = 1.51; *p* value  = 0.22; *I*_2_ (inconsistency) = 0% (95% CI 0–56.3%)] postoperative mortality between restrictive and liberal subgroups (Additional file 1: Figures 1–2).

#### Postoperative organ-related major complications and length of stay

**Fig. 4:**
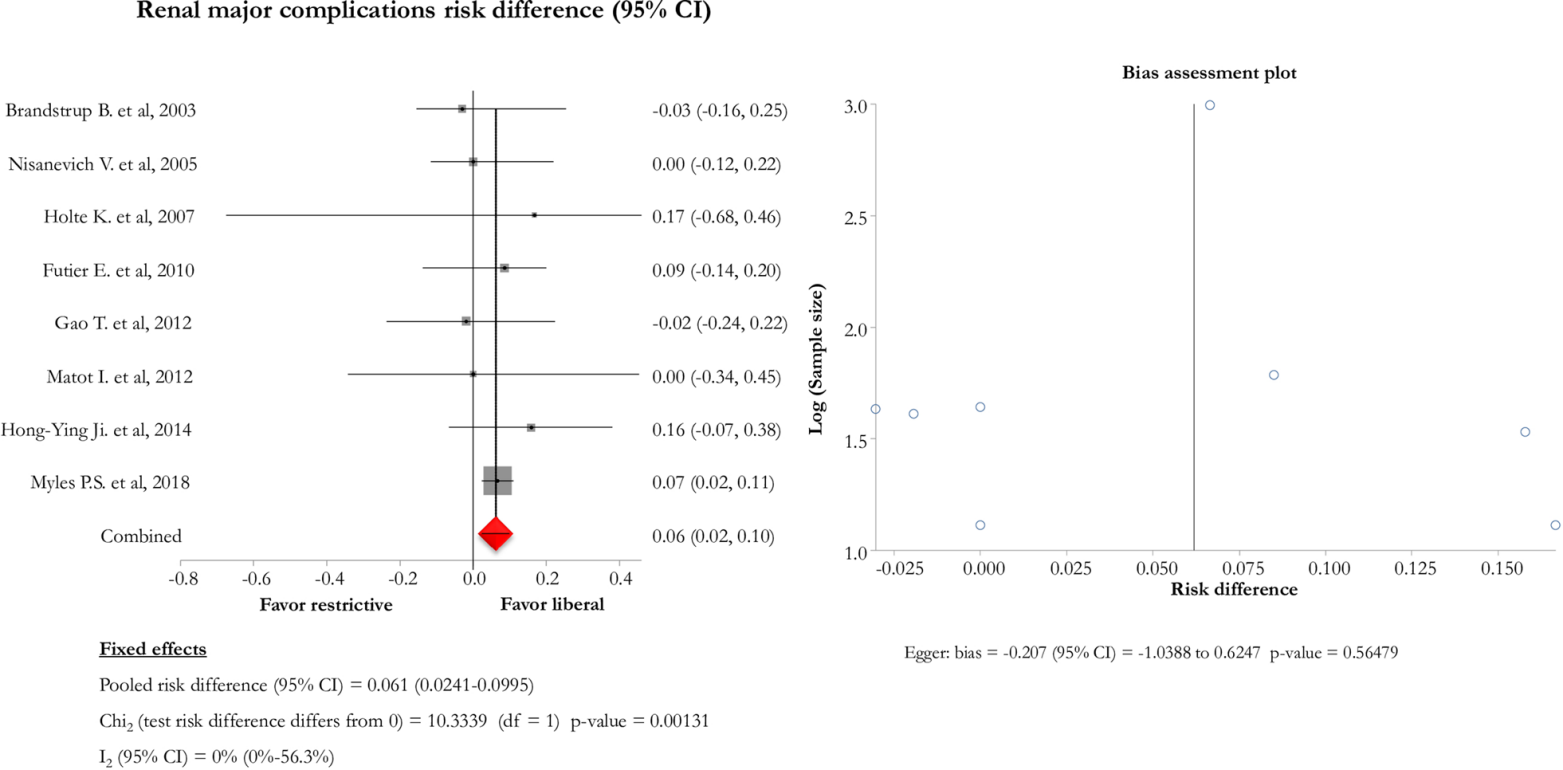
Forest and bias assessment plots of postoperative major renal events

Pooling data from the 8 studies, the liberal approach was associated with lower overall complication renal major events, as compared to the restrictive [pooled risk difference (95% CI) = 0.06 (0.02–0.09); Chi_2_ = 10.3; *p* value  = 0.001] (Fig. 4). In this subgroup, a sub-analysis regarding the use of type of fluid used intraoperatively (colloids *vs.* crystalloids) showed a borderline statistical significance regarding an increased incidence of major renal events in the restrictive populations receiving colloids, as compared to the liberal ones [mean (SD) major renal events 8.0% (4.7) vs. 1.7% (1.9); *p* = 0.05].

On the contrary, we found no difference between restrictive and liberal subgroups in the occurrence of either severe postoperative cardiovascular (9 studies; *p* value  = 0.88) or infective (10 studies; *p* value  = 0.10) complications (Additional file 1: Figures 3–4), or the length of hospital stay [7 days (6–9) *vs.* 7 days (5–8); *p* value  = 0.49].

### Subgroup analyses

**Fig. 5:**
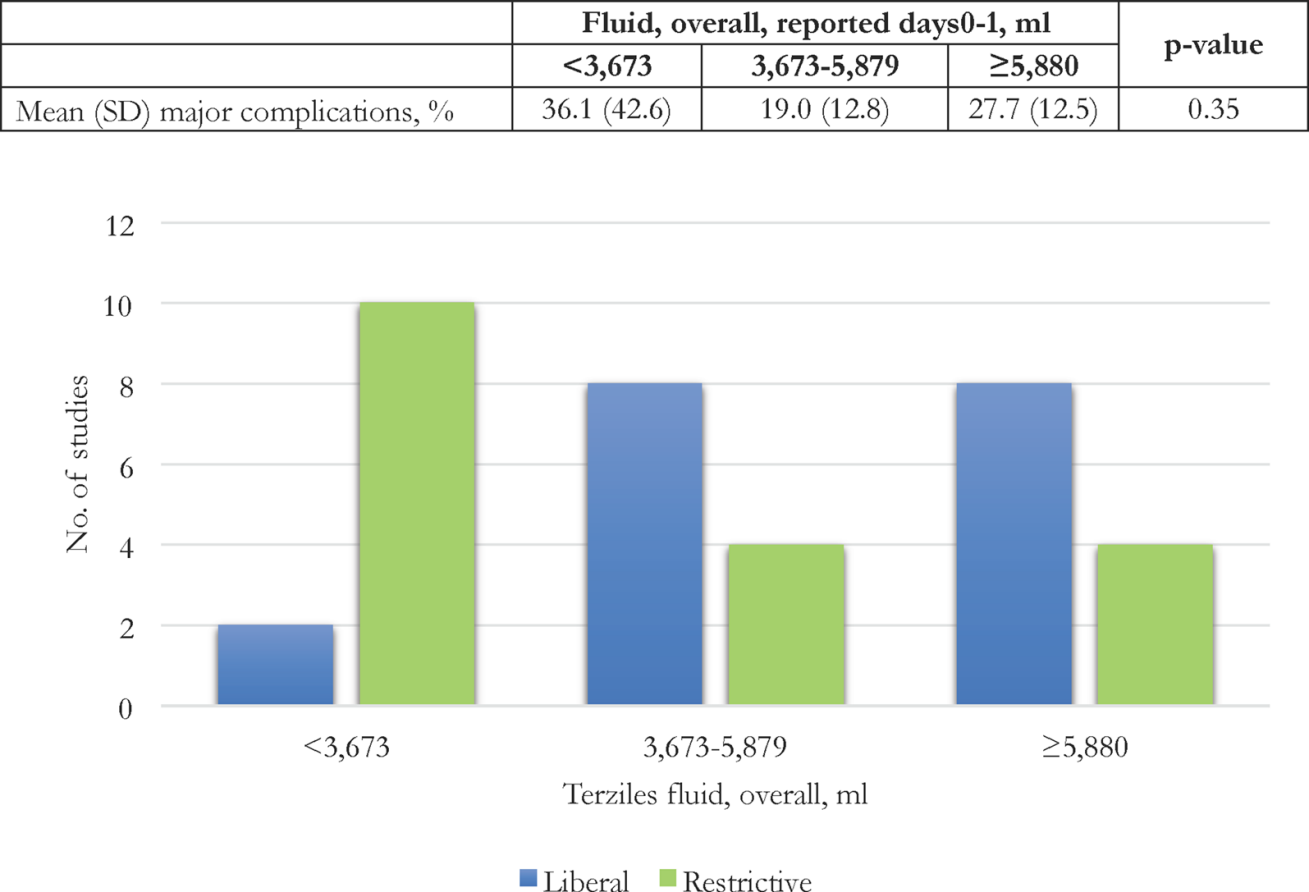
Liberal / restrictive classification of the subgroups of patients in the included studies. Data are reported according to the tertiles of overall fluid amount (in ml) reported in the days 0 (day of the operation) and 1 (first postoperative day)

As reported in Additional file 1: Table 5, preoperative ASA risk score did not impact the postoperative complications rate, among restrictive/liberal subgroups. No difference in the overall rate of major complications was found by stratifying the in tertiles of overall perioperative fluid administered (Fig. 5).

## Discussion

This metanalysis conducted on RCTs in major abdominal elective surgery regarding the effect of perioperative liberal or restrictive approach on postoperative outcomes found no difference between the two approaches in the occurrence of the overall major postoperative complications or mortality. On the contrary, the liberal fluid policy was associated with lower overall complication renal major events, as compared to the restrictive.

Postoperative complications are common after major surgery and represent a significant financial and social burden [2, 3]. Optimisation of fluid management has been extensively studied as a potentially modifiable perioperative factor by adopting specific protocols focused on modality of fluid administration and cumulative fluid balance [9–11, 40]. The analysis of the literature in this field is rather complex due to the number of variables potentially affecting the final outcomes, which includes the overall complexity and the intrinsic risks of each specific type of the surgery.

On the one hand, a tendency towards a more restrictive approach (as supported by the ERAS pathways [13]) has been reported. In fact, a previous metanalysis [19] showed that fewer patients had a lower total complication rate and risk of infection in the restrictive group as compared to the liberal group.

On the other, the recent large RCT performed by Myles et al. challenged this concept, showing that disability-free survival at 1 year did not change in patients randomised to a zero-balance *vs* a liberal approach, with the first strategy being associated with higher rates of acute kidney injury, surgical site infection and need for renal-replacement therapy [16]. Interestingly, this single trial enrolled more patients than 17 RCTs combined in the previous 15 years (Table 1). The weight of this trial specifically impacts on postoperative major renal events of the present metanalysis, which were lower in the liberal subgroup as compared to the restrictive (Fig. 4). Interestingly, renal events did not impact on overall mortality, despite the fact that acute kidney injury is recognised as an independent risk factor for mortality [41]. However, postoperative mortality is greatly affected by the results of the study of Myles et al. [16]. Moreover, the present metanalysis did not include trials on cardiac surgery, which is a clinical setting specifically associated with an increased risk of death in those patients who develop postoperative acute kidney injury [41–43].

Considering the impact of crystalloids/colloids use on the renal postoperative outcome, this metanalysis only suggests a possible additive effect of the colloids use in a restrictive approach. However, a recent large RCT comparing the use of low molecular weight hydroxyethyl-starch vs. 0.9% saline for intravascular volume expansion in high-risk surgical patients showed no significant difference in a composite outcome of death or significant postoperative [27]. For this reason, this result should be considered with extreme caution.

In the field of perioperative fluid administration, summarising the evidence available in the literature into straightforward clinical suggestions for daily clinical practice is rather complicated, and our updated results basically indicate an equivalence between the two perioperative fluid policies, suggesting a “third way”. The overall amount of fluid (perioperative target) should be integrated into an individualised goal-directed fluid replacement strategy (perioperative policy), to prevent fluid overload and fluid shortage by closely monitoring the effects of each bolus administered, as long as the individual plateau of the impact on predefined flow or pressure variables is achieved [12, 44]. Interestingly, this approach was incorporated into the intraoperative protocol of 5 studies [16, 25, 27–29], including the study of Myles et al. who assessed central venous pressure and stroke volume variation in case of intraoperative hypotension to guide resuscitation (supplementary appendix of the trial [16]). Moreover, a recent metanalysis showed a trend towards the reduction of postoperative complications when a goal-directed therapy is used in patients receiving large amounts of perioperative fluids [45]. Thus, rather than choosing between a fixed-volume regimen and a goal-directed concept, an alternative approach could be to combine the two strategies. Of note, in the study of Myles et al. [16] restrictive fluid therapy had similar effects in patients treated with or without a goal-directed device.

As shown by our qualitative analysis, neither the perioperative intakes nor the fluid balance or the bodyweight gain has been consistently reported in the considered RCTs. Surprisingly, the overall fluid balance or the postoperative weight difference, which should be, in principle, the most effective perioperative variables depicting the fluid paths of restrictive and liberal subgroups, has been reported only in 5 studies (27.7%) [16, 33, 35, 46, 47]. The heterogeneity in actual overall fluid balance computation, as long the definition of postoperative complications (see further) greatly affects the comparability of the RCTs, soliciting clear standards in data reporting.

Considering the fluid intake alone, our data suggest that an overall median administration of about 4 L on days 0 and 1 may be considered restrictive, whereas about 6 L may be considered liberal. However, as pointed out by previous metanalysis [18, 19], these cut-offs are associated with large inter-quartile ranges due to the lack of consistency in perioperative fluid regimen definition. Consequently, some subgroups of RCTs classified as restricted would be considered liberal in some other trials (see Fig. 5), because of overlapping perioperative volumes.

However, despite the lack of statistical significance, we found the highest rates of major complications in the highest and lowest tertiles of our sub-analysis.

Finally, the included studies all report overall low perioperative mortality rates; thus, as expected, the impact of liberal *vs.* restrictive strategies on perioperative mortality was not significant.

### Limitations

Some limitations of this study should be acknowledged.

First, as concern the data obtained from the included studies: 1) the definition of postoperative complications and the timing in mortality assessment may vary among the included studies, implying a bias in the reported outcomes’ comparability. In fact, some complications (*i.e.* pneumonia, wound abscess/infection/dehiscence, pneumothorax) have been considered either as minor or major, according to study-specific criteria, which have been respected in the data analysis; 2) we computed major/minor complications according to either the criteria adopted in the study or (only for subgroup analysis) definitions clearly indicating the gravity degree (*i.e.* peritonitis and anastomotic leak has been always considered as severe complications); 3) the impact of restrictive/liberal policies on specific subgroups of patients is overall lacking (*i.e.* patients with pre-existing renal or diastolic cardiac dysfunction), limiting the generalisability of data; 4) this is not a meta-analysis based on individual data and the main author of each study (whenever needed) has been contacted only to provide more specific information regarding the results on the overall population enrolled. This is specifically important for colloids/crystalloids sub-analysis.

Third, the overall quality of the included studies reported “some concerns” in the majority of them (55.5%), mostly related to the selection of the reported results due to drawbacks in the trial registration. This is mainly due to the criteria imposed by the RoB 2 scale [22] for the trial registration. The International Committee of Medical Journal Editors policy regarding prospective trial registration was started in 2005 [48], and most of the trial published before 2010 do not fulfil the RoB 2 criteria for “low-risk” report. Moreover, a monitoring of the study protocol is reported only in three studies [16, 26, 33].

Finally, we adopted a database combination search strategy, including PUBMED®, EMBASE® and the Cochrane Controlled Clinical trials register, excluding different sources (*i.e.* Web of Science®). Although this choice should allow a reliable coverage of the published studies for the topic of interest, some RCTs could not be identified.

## Conclusions

In major abdominal elective surgery perioperative, the choice between liberal or restrictive approach did not affect overall major postoperative complications or mortality. In a subgroup analysis, a liberal fluid policy was associated with lower overall complication renal major events, as compared to the restrictive.

The lack of consistency in perioperative overall fluid balance and in the definitions of clinical outcomes still affects the comparability of the results of RTCs, soliciting clear standards in data reporting.

## Supplementary Information

**Additional file 1**. Supplementary materials including supplementary tables and figures.

## Data Availability

The datasets used and/or analysed during the current study are available from the corresponding author on reasonable request.

## Abbreviations

ASA: American Society of Anaesthesiologists (ASA) physical status score
RCT: Randomised controlled trial
ERAS: Enhanced recovery after surgery
SD: Standard deviation
95%CI: 95% Confidence intervals
