# Supplementary material for: Perioperative liberal versus restrictive fluid strategies and postoperative outcomes: a systematic review and metanalysis on randomised-controlled trials in major abdominal elective surgery
Source: Crit Care. 2021 Jun 11;25:205. doi: 10.1186/s13054-021-03629-y (PMC8194047; doi:10.1186/s13054-021-03629-y)
Supplement: Supplementary file 1 — Additional file 1. Supplementary materials including supplementary tables and figures. [file 13054_2021_3629_MOESM1_ESM.docx]

**Perioperative liberal versus restrictive fluid strategies and postoperative outcomes: a systematic review and metanalysis on randomised-controlled trials in major abdominal elective surgery.**

Informative title: perioperative fluid strategies and postoperative outcomes

Antonio Messina, MD, PhD^1,2^; Chiara Robba, MD, PhD^3^; Lorenzo Calabrò, MD^1^; Daniel Zambelli, MD^1^; Francesca Iannuzzi, MD^3,4^; Edoardo Molinari, MD^3,4^; Silvia Scarano, MD^3,4^; Denise Battaglini, MD^3^; Marta Baggiani, MD^5^; Giacomo De Mattei^6^; Laura Saderi, BsC^7^; Giovanni Sotgiu MD, PhD^7^; Paolo Pelosi, MD, FERS^3,4^; Maurizio Cecconi MD, FRCA, FICM^1,2^.

^1^Humanitas Clinical and Research Center – IRCCS, Rozzano, MI, Italy; ^2^Department of Biomedical Sciences, Humanitas University, Pieve Emanuele, MI, Italy; ^3^Anaesthesia and Intensive Care, San Martino Policlinico Hospital, IRCCS for Oncology and Neuroscience, Genoa, Italy; ^4^Department of Surgical Sciences and Integrated Diagnostic (DISC), University of Genoa, Genoa, Italy; ^5^Anesthesia and Intensive Care Medicine, Maggiore della Carità University Hospital, Novara, Italy; ^6^Anesthesia and Intensive Care Medicine, Azienda Sanitaria Universitaria Integrata Udine, Italy; ^7^Clinical Epidemiology and Medical Statistics Unit, Departement of Medical, Surgical and Experimental, University of Sassari

**Corresponding author:**

Antonio Messina; Department of Anaesthesia and Intensive Care Medicine

Humanitas Clinical and Research Center – IRCCS

Via Alessandro Manzoni, 56

20089 – Rozzano (MI) - Italy

Email: [antonio.messina@humanitas.it](mailto:antonio.messina@humanitas.it)

**SUPPLEMENTAL MATERIALS**

**Table of Contents**

**Supplemental Table 1** PRISMA-DTA checklist.

**Supplemental Table 2** Extracted data in each study assessed for eligibility.

**Supplemental Table 3** Full text articles excluded, not fitting eligibility criteria.

**Supplemental Table 4** Intraoperative haemodynamic protocol and postoperative care.

**Supplemental Figure 1** Forest and bias assessment plots of early postoperative mortality

**Supplemental Figure 2** Forest and bias assessment plots of late postoperative mortality

**Supplemental Figure 3** Forest and bias assessment plots of major cardiovascular complications.

**Supplemental Figure 4** Forest and bias assessment plots of major infective complications.

**Supplemental Table 1. PRISMA-DTA checklist for systematic review and meta-analysis**

| **Section/topic** | **#** | **Checklist item** | **Reported on page #** |
| --- | --- | --- | --- |
| **TITLE** | | |  |
| Title | 1 | Identify the report as a systematic review, meta-analysis, or both. | 1 |
| **ABSTRACT** | | |  |
| Structured summary | 2 | Provide a structured summary including, as applicable: background; objectives; data sources; study eligibility criteria, participants, and interventions; study appraisal and synthesis methods; results; limitations; conclusions and implications of key findings; systematic review registration number. | 3-4 |
| **INTRODUCTION** | | |  |
| Rationale | 3 | Describe the rationale for the review in the context of what is already known. | 5 |
| Objectives | 4 | Provide an explicit statement of questions being addressed with reference to participants, interventions, comparisons, outcomes, and study design (PICOS). | 6 |
| **METHODS** | | |  |
| Protocol and registration | 5 | Indicate if a review protocol exists, if and where it can be accessed (e.g., Web address), and, if available, provide registration information including registration number. | 7 |
| Eligibility criteria | 6 | Specify study characteristics (e.g., PICOS, length of follow-up) and report characteristics (e.g., years considered, language, publication status) used as criteria for eligibility, giving rationale. | 7 |
| Information sources | 7 | Describe all information sources (e.g., databases with dates of coverage, contact with study authors to identify additional studies) in the search and date last searched. | 7-8 |
| Search | 8 | Present full electronic search strategy for at least one database, including any limits used, such that it could be repeated. | 7-8 |
| Study selection | 9 | State the process for selecting studies (i.e., screening, eligibility, included in systematic review, and, if applicable, included in the meta-analysis). | 8 |
| Data collection process | 10 | Describe method of data extraction from reports (e.g., piloted forms, independently, in duplicate) and any processes for obtaining and confirming data from investigators. | 8 |
| Data items | 11 | List and define all variables for which data were sought (e.g., PICOS, funding sources) and any assumptions and simplifications made. | 9-10 |
| Risk of bias in individual studies | 12 | Describe methods used for assessing risk of bias of individual studies (including specification of whether this was done at the study or outcome level), and how this information is to be used in any data synthesis. | 9-10 |
| Summary measures | 13 | State the principal summary measures (e.g., risk ratio, difference in means). | 9-10 |
| Synthesis of results | 14 | Describe the methods of handling data and combining results of studies, if done, including measures of consistency (e.g., I^2^) for each meta-analysis. | 9-10 |

Page 1 of 2

| **Section/topic** | **#** | **Checklist item** | **Reported on page #** |
| --- | --- | --- | --- |
| Risk of bias across studies | 15 | Specify any assessment of risk of bias that may affect the cumulative evidence (e.g., publication bias, selective reporting within studies). | 11 |
| Additional analyses | 16 | Describe methods of additional analyses (e.g., sensitivity or subgroup analyses, meta-regression), if done, indicating which were pr Supplemental specified. | 9-10 |
| **RESULTS** | | |  |
| Study selection | 17 | Give numbers of studies screened, assessed for eligibility, and included in the review, with reasons for exclusions at each stage, ideally with a flow diagram. | 11-12 |
| Study characteristics | 18 | For each study, present characteristics for which data were extracted (e.g., study size, PICOS, follow-up period) and provide the citations. | 11-12 |
| Risk of bias within studies | 19 | Present data on risk of bias of each study and, if available, any outcome level assessment (see item 12). | 11-12 |
| Results of individual studies | 20 | For all outcomes considered (benefits or harms), present, for each study: (a) simple summary data for each intervention group (b) effect estimates and confidence intervals, ideally with a forest plot. | 11-12 |
| Synthesis of results | 21 | Present results of each meta-analysis done, including confidence intervals and measures of consistency. | 11-12 |
| Risk of bias across studies | 22 | Present results of any assessment of risk of bias across studies (see Item 15). | 11-12 |
| Additional analysis | 23 | Give results of additional analyses, if done (e.g., sensitivity or subgroup analyses, meta-regression [see Item 16]). | 11-12 |
| **DISCUSSION** | | |  |
| Summary of evidence | 24 | Summarize the main findings including the strength of evidence for each main outcome; consider their relevance to key groups (e.g., healthcare providers, users, and policy makers). | 13-14 |
| Limitations | 25 | Discuss limitations at study and outcome level (e.g., risk of bias), and at review-level (e.g., incomplete retrieval of identified research, reporting bias). | 15-16 |
| Conclusions | 26 | Provide a general interpretation of the results in the context of other evidence, and implications for future research. | 17 |
| **FUNDING** | | |  |
| Funding | 27 | Describe sources of funding for the systematic review and other support (e.g., supply of data); role of funders for the systematic review. | 18 |

**Supplemental Table 2. Extracted data in each study assessed for eligibility.**

| Study Reference | Names and surnames of authors, year of publication. |
| --- | --- |
| Type of surgery | Surgical setting where the study has been performed |
| Patients’ characteristics and peri-operatory risk. | Male/Female ratio; age; Body mass index; height; weight (as mean ± sd or median). American Society of Anesthesiologists physical status. |
| Patients’ enrollment | Number of enrolled/eligible/dropped out patients, in the two study groups |
| Peri-operatory fluids | Cumulative Intraoperative, postoperative (day 0 and 1), overall fluid volume infusions (colloids or crystalloids), as mean ± sd or median (interquartiles). Oral intake. |
| Mortality | Mortality rate reported with definition. |
| Complications | Expressed as overall number of events reported as percentage of patients who had at least one complication |
| Organ-specific complications | Cardiac, renal, infections, pulmonary, neurological, hemorrhagic, other. |

**Supplemental Table 3. Full-text articles excluded, not fitting eligibility criteria.**

| **Excluded Studies** | | **Reason for exclusion** | |
| --- | --- | --- | --- |
| Fischer et al. Ann Surg 2009 | | Normovolemic hemodilution protocol | |
| Lobo et al. Lancet 2002 | | < 15 patients per group | |
| McArdle et al. Ann Surg 2009 | | < 15 patients per group | |
| Peng et al. Hepatogastroenterology 2013 | | Pdf file not available | |
| Gonzalez-Fajardo et al. Eur J Vasc Endovasc Surg 2009 | | No difference in volume received by the two groups | |
| Vermeulen et al. Trials 2010 | | No difference in volume received by the two groups | |
| Hubner et al. Journal of Surgical Research 2012 | | Fast-track vs. standard care trial. | |
| Holte et al. [Ann Surg](https://www.ncbi.nlm.nih.gov/pmc/articles/PMC1356497/). 2004 | | Complication rate not reported | |
| Matot et al. 2013 | | Thoracic surgery | |
| Lobo et al 2011 | | Not only abdominal surgery | |
|  |  | |  |

**Supplemental Table 4.** Intraoperative haemodynamic protocol and postoperative care.

| **Study** | **Hemodynamic intraoperative**  **protocol** | **Hemodynamic**  **trigger** | **GDT treatment** | PACU | | ICU | | Recovery  room | |
| --- | --- | --- | --- | --- | --- | --- | --- | --- | --- |
|  |  |  |  | **RES** | **LIB** | **RES** | **LIB** | **RES** | **LIB** |
| Brandstrup B.[1] | NO | - | - | - | - | - | - | ** | ** |
| Kabon B.[2] | NO | - | - | - | - | 2.4% | 6.2% | - | - |
| Nisanevich V.[3] | YES | HR/ABP | Intra-operative treatment of tachycardia (heart rate > 90 beats/min or > 20% above baseline) accompanied by low blood pressure (< 90 mmHg or < 20% below baseline) was treated with a bolus of 250 mL Ringer Lactate over 15 minutes repeated up to a maximum of 1500 mL if necessary. | - | - | - | - | ** | ** |
| Holte K.[4] | NO | - | - | 93 min**  (55 – 575) | 113**  (60 – 450) | - | - | - | - |
| Muller S.[5] | NO | - | - | NA | NA | NA | NA | NA | NA |
| Futier E.[6] | YES | PPV | PPV > 13% was corrected with a 250-mL colloid bolus. The fluid challenge was repeated, if necessary, until PPV was corrected and there was no further increase in stroke volume. | ** | ** | - | - | - | - |
| Gao T.[7] | NO | - | - | - | - | - | - | ** | ** |
| Matot I.[8] | YES | HR/ABP | Fluid boluses (250 mL Ringer Lactate) when low blood pressure (<90 or >20% below baseline) on 2 consecutive measurements | ** | ** | - | - | - | - |
| Abraham-Nordling M.[9] | NO | - | - | NA | NA | NA | NA | NA | NA |
| Kalyan JP.[10] | NO | - | - | NA | NA | NA | NA | NA | NA |
| Lavu H.[11] | NO | - | - | - | - | ** | ** | - | - |
| Hong-Ying J.[12] | NO | - | - | NA | NA | NA | NA | NA | NA |
| Piljic D.[13] | NO | - | - | - | - | ** | ** | - | - |
| van Samkar G.[14] | NO | - | - | ** | ** | - | - | - | - |
| Grant F.[15] | NO | - | - | ** | ** | - | - | - | - |
| Kassim D.[16] | YES | SaO_2_ - ScVO_2_ | The management protocol was to keep O_2_ER< 27% and checked hourly; if exceeded 27% check for CVP; if <10 cmH2O and haematocrit >30%, a fluid challenge of 250 ml of colloids was given (or blood transfusion when haematocrit <30%). If O_2_ER still >27%, dobutamine infusion (3-15 micrograms/kg/min) was started. But if CVP >10 cmH_2_O, dobutamine infusion started without fluid challenge | - | - | ** | ** | - | - |
| Myles P.S.[17] | YES | CVP/SVV | SVV > 10% triggered a bolus of 3 ml/kg of fluids. The occurrence of hypotension (systolic blood pressure < 90 mmHg) was treated according to CVP (different threshold for liberal and restrictive groups) and SVV values (≥ 13% or not). | - | - | 27.9%* | 28.0%* | - | - |
| Wuethrich P.Y.[18] | NO | - | - | NA | NA | NA | NA | NA | NA |

HR, heart rate; ABP, arterial blood pressure; PPV, pulse pressure variation; SV, stroke volume; SaO_2_; arterial oxygen saturation; ScVO_2_; central venous oxygen saturation; O_2_ER, oxygen extraction; SVV, stroke volume variation; FTC, doppler corrected flow time; CVP, central venous pressure; PACU, post-anaesthesia care unit; ICU, intensive care unit; NA, data not available. RES/LIB refers to those patients who received a restrictive or liberal strategy, respectively.

** = all patients admitted to PACU/ICU after the surgery

* = planned admission to ICU

**Supplemental Table 5.** Overall rate of complications according to preoperative risk score.

|  | **Restrictive group** | **Liberal group** | **Differences**  **(95% CI)** | **p-value** |
| --- | --- | --- | --- | --- |
| **ASA I-II ≥ 50%** | *Mean (95% CI)* | *Mean (95% CI)* |  |  |
| *Overall complications, %* | 26.9 (12.1; 41.6) | 35.6 (21.9; 49.2) | -8.7 (-27.1; 9.6) | 0.33 |
| **ASA III-IV ≥ 50%** | **Restrictive group** | **Liberal group** |  |  |
| *Overall complications, %* | 23.7 (-22.1; 69.5) | 37.1 (-5.4; 79.6) | -13.4 (-53.7; 26.9) | 0.41 |

ASA, American Society of Anesthesiologists physical status score.

**Supplemental Figure 1 – Forest and bias risk assessment plots of early postoperative mortality (≤ 30 postoperative days)**

**
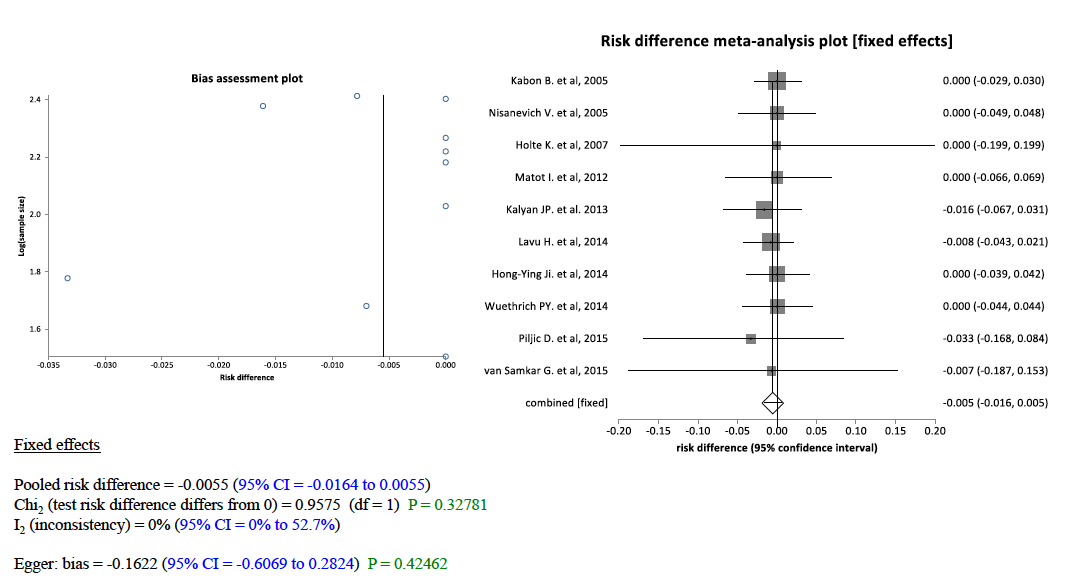
**

**Supplemental Figure 2 – Forest and bias risk assessment plots of late postoperative mortality (> 30 postoperative days).**

**
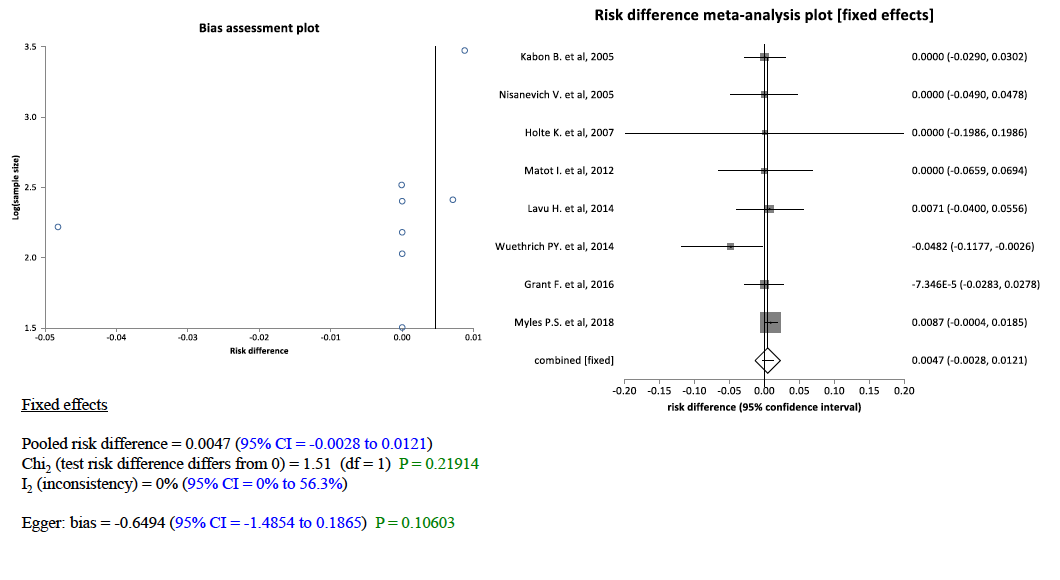
**

**Supplemental Figure 3 – Forest and bias risk assessment plots of major cardiovascular complications.**

**
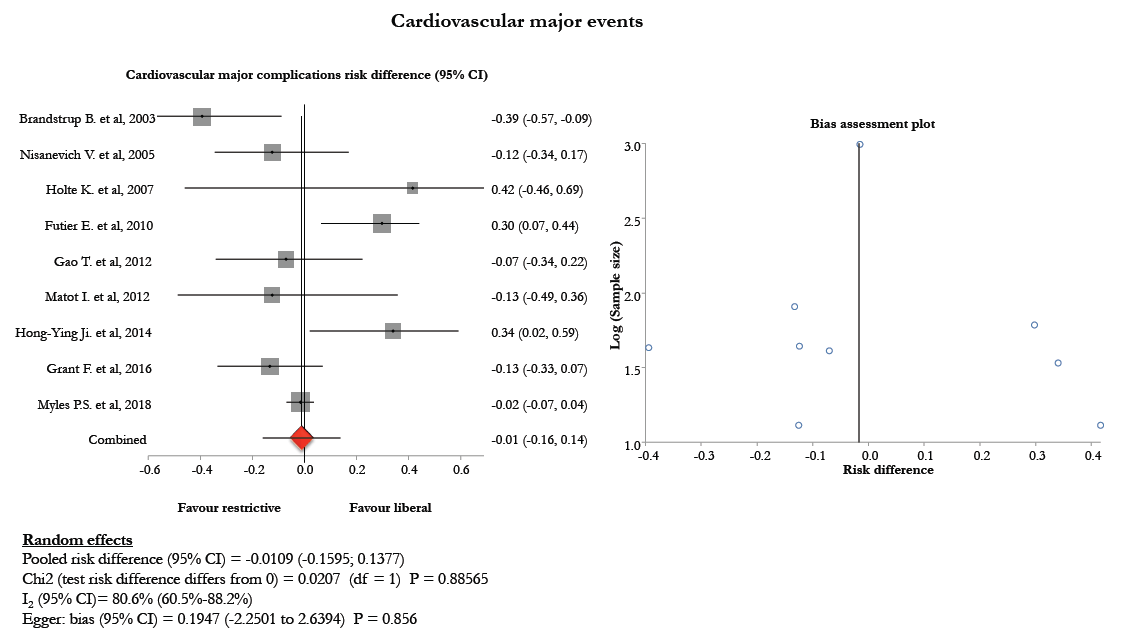
**

**Supplemental Figure 4 – Forest and bias risk assessment plots of major infective complications.**

**
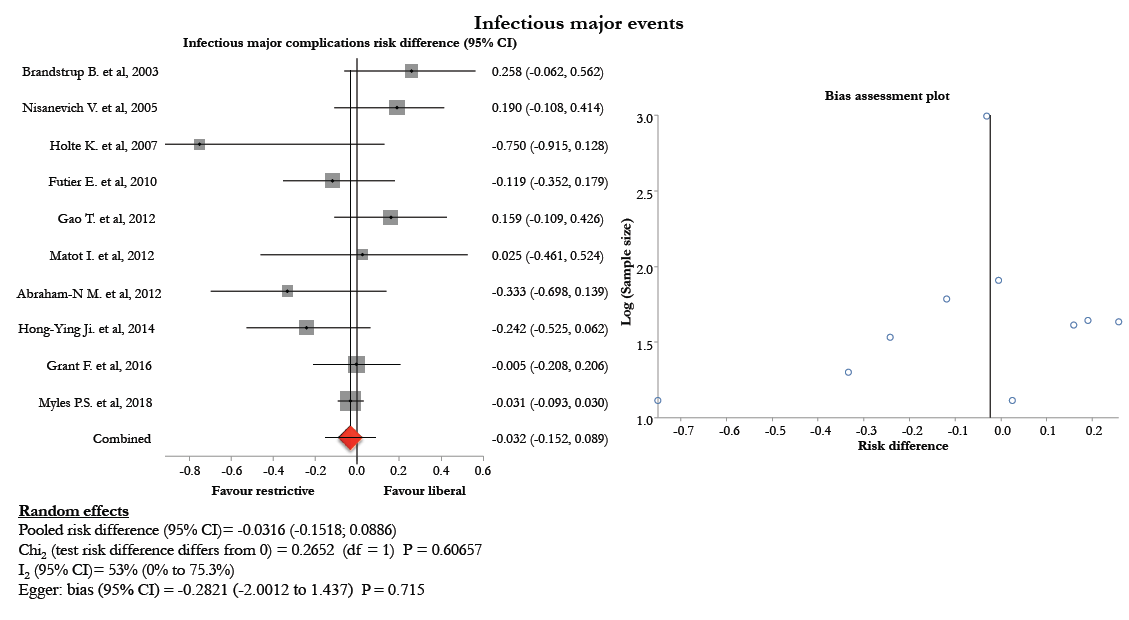
**

**Bibliography of Supplemental Materials**

1 Brandstrup B, Tonnesen H, Beier-Holgersen R, Hjortso E, Ording H, Lindorff-Larsen K, et al.: Effects of intravenous fluid restriction on postoperative complications: Comparison of two perioperative fluid regimens: A randomized assessor-blinded multicenter trial. Ann Surg 2003;238:641-648.

2 Kabon B, Akca O, Taguchi A, Nagele A, Jebadurai R, Arkilic CF, et al.: Supplemental intravenous crystalloid administration does not reduce the risk of surgical wound infection. Anesth Analg 2005;101:1546-1553.

3 Nisanevich V, Felsenstein I, Almogy G, Weissman C, Einav S, Matot I: Effect of intraoperative fluid management on outcome after intraabdominal surgery. Anesthesiology 2005;103:25-32.

4 Holte K, Foss NB, Andersen J, Valentiner L, Lund C, Bie P, et al.: Liberal or restrictive fluid administration in fast-track colonic surgery: A randomized, double-blind study. Br J Anaesth 2007;99:500-508.

5 Muller S, Zalunardo MP, Hubner M, Clavien PA, Demartines N, Zurich Fast Track Study G: A fast-track program reduces complications and length of hospital stay after open colonic surgery. Gastroenterology 2009;136:842-847.

6 Futier E, Constantin JM, Petit A, Chanques G, Kwiatkowski F, Flamein R, et al.: Conservative vs restrictive individualized goal-directed fluid replacement strategy in major abdominal surgery: A prospective randomized trial. Arch Surg 2010;145:1193-1200.

7 Gao T, Li N, Zhang JJ, Xi FC, Chen QY, Zhu WM, et al.: Restricted intravenous fluid regimen reduces the rate of postoperative complications and alters immunological activity of elderly patients operated for abdominal cancer: A randomized prospective clinical trail. World J Surg 2012;36:993-1002.

8 Matot I, Paskaleva R, Eid L, Cohen K, Khalaileh A, Elazary R, et al.: Effect of the volume of fluids administered on intraoperative oliguria in laparoscopic bariatric surgery: A randomized controlled trial. Arch Surg 2012;147:228-234.

9 Abraham-Nordling M, Hjern F, Pollack J, Prytz M, Borg T, Kressner U: Randomized clinical trial of fluid restriction in colorectal surgery. Br J Surg 2012;99:186-191.

10 Kalyan JP, Rosbergen M, Pal N, Sargen K, Fletcher SJ, Nunn DL, et al.: Randomized clinical trial of fluid and salt restriction compared with a controlled liberal regimen in elective gastrointestinal surgery. Br J Surg 2013;100:1739-1746.

11 Lavu H, Sell NM, Carter TI, Winter JM, Maguire DP, Gratch DM, et al.: The hyslar trial: A prospective randomized controlled trial of the use of a restrictive fluid regimen with 3% hypertonic saline versus lactated ringers in patients undergoing pancreaticoduodenectomy. Ann Surg 2014;260:445-453; discussion 453-445.

12 Jie HY, Ye JL, Zhou HH, Li YX: Perioperative restricted fluid therapy preserves immunological function in patients with colorectal cancer. World J Gastroenterol 2014;20:15852-15859.

13 Piljic D, Petricevic M, Piljic D, Ksela J, Robic B, Klokocovnik T: Restrictive versus standard fluid regimen in elective minilaparotomy abdominal aortic repair-prospective randomized controlled trial. Thorac Cardiovasc Surg 2016;64:296-303.

14 van Samkar G, Eshuis WJ, Bennink RJ, van Gulik TM, Dijkgraaf MG, Preckel B, et al.: Intraoperative fluid restriction in pancreatic surgery: A double blinded randomised controlled trial. PLoS One 2015;10:e0140294.

15 Grant F, Brennan MF, Allen PJ, DeMatteo RP, Kingham TP, D'Angelica M, et al.: Prospective randomized controlled trial of liberal vs restricted perioperative fluid management in patients undergoing pancreatectomy. Ann Surg 2016;264:591-598.

16 I. KDE: Goal directed fluid therapy reduces major complications in elective surgery for abdominal aortic aneurysm: Liberal versus restrictive strategies. Egyptian Journal of Anaesthesia 2016;32:167-173.

17 Myles PS, Bellomo R, Corcoran T, Forbes A, Peyton P, Story D, et al.: Restrictive versus liberal fluid therapy for major abdominal surgery. N Engl J Med 2018;378:2263-2274.

18 Wuethrich PY, Burkhard FC, Thalmann GN, Stueber F, Studer UE: Restrictive deferred hydration combined with preemptive norepinephrine infusion during radical cystectomy reduces postoperative complications and hospitalization time: A randomized clinical trial. Anesthesiology 2014;120:365-377.
